# Predator size‐structure and species identity determine cascading effects in a coastal ecosystem

**DOI:** 10.1002/ece3.4571

**Published:** 2018-12-01

**Authors:** John N. Griffin, Brian R. Silliman

**Affiliations:** ^1^ Department of Biosciences Swansea University Swansea UK; ^2^ Division of Marine Science and Conservation, Nicholas School of the Environment Duke University Beaufort North Carolina

**Keywords:** biodiversity, ecosystem functioning, metabolic theory, multifunctionality, salt marsh, trophic cascade

## Abstract

Cascading consequences of predator extinctions are well documented, but impacts of perturbations to predator size‐structure and how these vary across species remain unclear. Body size is hypothesized to be a key trait governing individual predators' impact on ecosystems. Therefore, shifts in predator size‐structure should trigger ecosystem ramifications which are consistent across functionally similar species. Using a US salt marsh as a model system, we tested this hypothesis by manipulating size class (small, medium, and large) and size diversity (combination of all three size classes) within two closely related and functionally similar predatory crab species over 4 months. Across treatments, predators suppressed densities of a dominant grazer and an ecosystem engineer, enhanced plant biomass, and altered sediment properties (redox potential and saturation). Over the metabolically equivalent experimental predator treatments, small size class predators had stronger average impacts on response variables, and size class interacted with predator species identity to drive engineer suppression. Within both predator species, size diversity increased cannibalism and slightly weakened the average impact. These results show that predator impacts in a salt marsh ecosystem are determined by both size class and size diversity; they also highlight that size class can have species‐dependent and response‐dependent effects, underlining the challenge of generalizing trait effects.

## 1 INTRODUCTION

Human activities have driven functional, or total, extinctions of predators in many ecosystems (Dirzo et al., 2014; McCauley et al., 2015). Such perturbations often trigger trophic cascades that reach the base of food webs and have the potential to widely affect ecosystem properties (Duffy, 2003). Anthropogenic drivers, including climate change (Gardner, Peters, Kearney, Joseph, & Heinsohn, 2011) and size‐selective harvesting (Lester et al., 2009), are changing the size‐structure of predator populations. But, relative to species declines or extinctions, the potential ecosystem effects of these subtler perturbations to functional diversity have received little attention.

Body size is considered a key trait and potentially drives multiple aspects of predators’ functional ecology (Woodward et al., 2005). As examples, size increases per capita metabolic demand and ingestion rates (following ~0.7 power law; Kleiber, 1932, Brose, 2010), and may govern the strength and distribution of trophic interactions (Emmerson & Raffaelli, 2004). Furthermore, size may affect predators' capacity to physically engineer the environment through foraging or lair excavation (Solan et al., 2004). Reducing the dominant size class (or mean size) in a predator population may thus broadly influence prey and ecosystem‐level properties through multiple pathways and, accordingly, multifunctionality, that is, the simultaneous provision of multiple ecosystem functions (Byrnes et al., 2014; Duffy, Richardson, & Canuel, 2003). However, smaller predators tend to be more abundant (Damuth, 1981), potentially helping to compensate for weaker per capita effects and providing ecosystems a degree of functional resistance to size shifts. Although observational fisheries data indicate that reducing the mean size of a predator can indeed induce trophic cascades (Shackell, Frank, Fisher, Petrie, & Leggett, 2010), experimental tests have yielded mixed results (Jochum, Schneider, Crowe, Brose, & O'Gorman, 2012; McElroy et al., 2015; Rudolf & Rasmussen, 2013a, 2013b ), and studies have yet to identify consequences for ecosystem multifunctionality.

Body size should theoretically predict the ecological impacts of size classes within predator species, irrespective of species identity (e.g., Petchey & Belgrano, 2010). But variation in other traits across species boundaries may overwhelm or modify the effects of body size, diminishing its predictive capacity (Emmerson & Raffaelli, 2004; Rudolf, Rasmussen, Dibble, & Allen, 2014). For example, interspecific differences in foraging mode (roaming vs. sit and wait), rather than size, determine cascading ecosystem effects of spiders in grassland food webs (Schmitz, 2008). Individual body size, and by extension size‐structure, is, then, perhaps most likely to reliably explain predator effects within species that are otherwise functionally redundant, that is, whose functional traits are standardized. Even then, unmeasured or unknown traits might vary across species, confounding trait‐based approaches and potentially re‐emphasizing the role of species identity in ecosystem functioning.

Although previous studies of predator size‐structure have largely focused on shifts in mean size, shifts in the variance (or diversity) of predator body size may also exert cascading effects. Size diversity might have consequences analogous to those of species diversity, which tends to strengthen prey suppression and trophic cascades through niche complementarity (e.g., Northfield, Snyder, Ives, & Snyder, 2010). Conversely, increasing size ratios also commonly enhance intraguild predation (IGP), cannibalism, and interference (Griffen & Byers, 2006; Krenek & Rudolf, 2014), implying that size diversity may decrease prey suppression, resulting cascades and thus predator impacts on multiple ecosystem properties. Notably, although IGP or cannibalism may reduce predator density (Griffen & Byers, 2006), it also reduces predator activity and may therefore cause density‐independent effects on prey suppression (Krenek & Rudolf, 2014). In contrast to the two dozen experiments that have manipulated predator species diversity (reviewed by Griffin, Byrnes, & Cardinale, 2013), only a few have manipulated predator size diversity (i.e., three or more size classes) (Rudolf, 2012; Toscano & Griffen, 2012) and these have reported variable effects on short‐term prey consumption rates. The trophic cascading and broader ecosystem effects of size diversity, and the consistency of these effects across species, therefore remain largely unknown.

To better understand the relative impacts of predator body size, size diversity and taxonomic identity on ecosystem functioning, we experimentally manipulated these predator variables, using a southeastern U.S. salt marsh as a model system. These marshes are characterized by vast stands of smooth cordgrass, *Spartina alterniflora*, and a relatively simple food web with strong trophic feedbacks. We manipulated the system's two species of resident, predatory crab (*Eurytium limosum* and *Panopeus obesus*) which are closely related and occur within the mud crab (Panopeidae) family (see Griffen & Mosblack, 2011 for phylogeny). Consequently, they are also functionally similar, sharing sit‐and‐wait hunting modes, benthic/burrow microhabitats, and body size ranges. Although these predators are known to regulate populations of grazing snails and ecosystem engineering fiddler crabs (Griffin, Toscano, Griffen, & Silliman, 2015), it is not known how they simultaneously affect sediment and plant properties, or how size and size diversity in these predator populations alter these multifunctional impacts. Based on the hypothesis that predator size is an important functional trait determining the trophic and non‐trophic interactions of predators, we tested the following predictions in our field experiment: (a) mean size (i.e., size class) will govern the multifunctional impacts of predators; (b) size diversity will foster cannibalism, reduce predator survivorship, and weaken these impacts; and, given the trait similarity of predator species, (c) the effects of size class and diversity on collective functional impacts will hold across species.

## 2 MATERIALS AND METHODS

We conducted our field experiment at Dean Creek salt marsh (31 23′N 81 16′W) on Sapelo Island, Georgia, USA. This site is dominated by *Spartina,* typical of salt marshes along the southeastern U.S. coast. *Eurytium* and *Panopeus* occur at aggregate densities of up to 15 individuals per square meter (J. N. Griffin, unpublished data), with individuals of a range of sizes (<2 mm to ~45 mm) of both species occurring in spatiotemporally well‐mixed populations (Silliman, Layman, Geyer, & Zieman, 2004). Our experimental design included the factor “size‐structure” which incorporated treatments of varying predator size classes (to test Prediction 1 [P1]) and predator size diversity (to test P2). We orthogonally manipulated size‐structure (small, medium, large, diverse) and species identity (*Eurytium*,* Panopeus*; to test P3), with the addition of a predator‐free control yielding a total of nine treatments. These treatments were replicated eight times (72 in total) and randomly assigned to field enclosures. After four months (4 June–4 October 2010), we examined densities of the functionally important prey and measured several ecosystem properties.

Field enclosures (0.7 m × 0.7 m) consisted of a pine frame with galvanized wire mesh (8 mm) panels stapled on all sides and the top. They were 100 cm in height aboveground, extended a further 35 cm belowground, and had a 5 cm wide, 35 cm deep inner border of crushed oyster shell to prevent escape of burrowing crabs. Enclosures were installed on small natural mounds of ribbed mussels (*Geukensia demissa*), habitat patches that naturally host high densities of mud crabs (Angelini et al., 2015), and were separated by >2 m and spanned a ~125 m × ~5 m band approximately equidistant to a tributary creek. Monocultures of intermediate height *Spartina* naturally occurred in all enclosures. We set initial densities of grazing snails, *Littoraria irrorata*, to 150 ind. enclosure^−1^ (adult, 15–17 mm length), a naturally occurring intermediate density (Silliman & Zieman, 2001). Declines in *Littoraria* densities in the predator‐free controls (average of 23% each month) were observed, possibly due to extreme heat stress at this site in the summer months. To compensate for non‐predatory losses, we re‐established starting densities in predator‐free controls and added equivalent snail numbers (equal to the mean added to controls) to all other treatments each month. Fiddler crabs, *Uca pugnax*, are infaunal and thus initial densities could not be feasibly equalized, but this species was relatively evenly distributed across field enclosures and was included in the experiment at ambient naturally occurring densities (49.00 ± 14.64 [mean ± *SD*] ind. enclosure^−1^). There were no initial differences among treatments in *Spartina* height or stem density and abundance of *Uca* or *Geukensia* (*p* > 0.45 in all cases).

Three predator size categories were defined based on carapace width: small (20–24 mm), medium (28–32 mm), and large (36–40 mm), which were used to create the four experimental treatments for each species. We adjusted the density of crabs to approximately equalize metabolic biomass (mass^0.75^) across treatments (Chalcraft & Resetarits, 2004; Schmitz & Price, 2011). Note that both species conformed to the same carapace width–body mass relationship (ANCOVA: width*identity, *p* > 0.7). We estimated metabolic biomass of mid‐sized crabs within each size class (i.e., small = 2.73 g, medium = 5.43 g, and large = 9.19 g), yielding approximate metabolic equivalence ratios of 1 large: 2 medium: 3 small. These ratios were maintained across size‐structure treatments by establishing the following densities in respective treatments (ind. enclosure^−1^): small = 9; medium = 6; large = 3; diverse = (3 × small) + (2 × medium) + (1 × large), applied to both species. The higher relative abundance of smaller predators maintains metabolic equivalence (Damuth, 1981) and is consistent with observational data in our study system (Silliman et al., 2004). A substitutive design was followed across the single and diverse treatments. See Supporting Information Appendix S1 for a schematic of the experimental design.

Predatory crabs were collected by hand from burrows in the field and assigned to treatments based on size class and species identity. The proportion of males varied with crab size class and species identity (Supporting Information Appendix S2). Because individuals were collected unselectively, sex ratios were considered representative of the natural populations. (Notably, all of our findings were qualitatively insensitive to inclusion of predator sex ratio as a model covariate). We checked each enclosure every 14 days throughout the experiment and compensated for losses of predators due to cannibalism or other causes of death. This density compensation ensured that the outcome of the experiment was not dominated by one‐off predation events in the small populations within cages; additionally, it of course results in conservative estimates of density‐dependent effects of predator interactions (since densities were only temporarily reduced) while leaving density‐independent effect unchanged. At each of the seven biweekly checks, we tallied the number of predatory crabs that were present and alive in each enclosure. To maintain treatment integrity, we also removed and replaced any crabs that had outgrown their size class (27 ind. over the experiment, 7% of those initially present).

We quantified prey, plant, and sediment response variables at the end of the experiment. To quantify the final abundance of *Littoraria*, all individuals were removed before counting. Removal of burrowing *Uca* was infeasible; we thus quantified a proxy of density by counting the total number of distinctive *Uca* burrows per enclosure (Bertness, 1985; Holdredge, Bertness, Herrmann, & Gedan, 2010). Small (<8 mm) individual *Uca* were able to move into and out of field enclosures, and thus, the final densities of this species include net effects of immigration and emigration of small individuals. To enumerate the linear leaf scarring (hereafter “leaf scars”) by the grazers (Silliman & Zieman, 2001), we visually estimated the lengths of all scars on all leaves of 10 *Spartina* stems in each enclosure and calculated the mean total length of scars per leaf. Finally, we quantified aboveground *Spartina* biomass through complete destructive sampling and oven‐drying (at 60 degrees until stable mass), and belowground *Spartina* biomass by extracting a 10 cm × 30 cm (diameter × depth) core from each plot, washing, and oven‐drying belowground biomass. Sediment redox potential has implications for biogeochemical processes (e.g., Gribsholt, Kostka, & Kristensen, 2003) and was measured by placing a redox probe (Hach Lange™ multi‐probe) 5 cm into the substrate at six haphazardly chosen locations in each field enclosure. To work out sediment saturation, a measure of the drainage and an additional determinant of biogeochemical processes (e.g., Hackney, 1987), we took a single 12 × 15 cm (diameter × depth) core from a randomly selected location (avoiding mud crab burrows) in each plot, before weighing it wet and oven‐dried to calculate % water mass. For all prey, plant, and sediment variables, we calculated the total predator effect (PE_t_), as: Ln(+pred/−pred), where +pred is the observed value in a predator‐containing enclosure, and −pred is the mean value in the predator‐free controls (Berlow, 1999). PE_t_ is an interaction strength metric, indicating the natural logarithm of predators’ proportional effects on the response variables.

### 2.1 Analysis

Statistical models detailed below included size‐structure (small, medium, large, diverse), species identity (*Eurytium*,* Panopeus*), and their interaction as fixed factors. Effects on predator survivorship were evaluated using a generalized linear mixed model (GLMM), with binomial errors and enclosure as a random effect (to account for multiple non‐independent measures per enclosure through time), in the lme4 package (Bates, Maechler, Bolker, & Walker, 2015) of R (R Core Team 2014). Effects on prey, plant, and sediment PE_t_ were assessed using two‐way analysis of variance (ANOVA) with Type III sum of squares in SPSS 22.0 (SPSS Inc., Chicago, Illinois). Inferences regarding the effect of predator presence, size class (P1), and size diversity (P2) were based on, respectively, the model intercept (grand mean of PE_t_), post hoc Tukey tests (to elucidate whether differences occurred between single size class treatments), and planned linear contrasts (between pooled single size class treatments and the diverse treatments). *p*‐Values across ANOVAs and linear contrasts were adjusted to control the false discovery rate (FDR; Benjamini & Hochberg, 1995).

Following recent research on ecosystem multifunctionality (Byrnes et al., 2014), we also calculated a measure of predator impact on the integrated suite of prey, plant, and sediment response variables. We worked with variables in their raw form, rather than total predator effects, and prepared the data as follows. First, we inverted (multiplied by −1) values of sediment redox, so that greater positive values indicated a stronger predator impact. Second, we scaled each response variable as a proportion of its mean five highest values (Byrnes et al., 2014). Third, for responses where lower positive values are indicative of stronger predator effects (e.g., prey density), we subtracted proportions from 1. Finally, we calculated the average values of these proportions across all responses, that is, average predator impact (API). We tested size‐structure and species identity effects on API (bounded between 0 and 1) based on beta regression with a log‐link using the betareg package (Cribari‐Neto & Zeileis, 2010) in R. *p*‐values were not directly available for factors using beta regression, so inferences were based on ΔAIC after dropping individual factors. To test P1 and P2, we used linear contrasts, with FDR adjusted *p*‐values, implemented in the R package multcomp (Hothorn, Bretz, & Westfall, 2008). In all cases, models were validated by ensuring there was not excessive heterogeneity of residuals.

## 3 RESULTS

### 3.1 Size‐structure and predator survivorship

**Figure 1:**
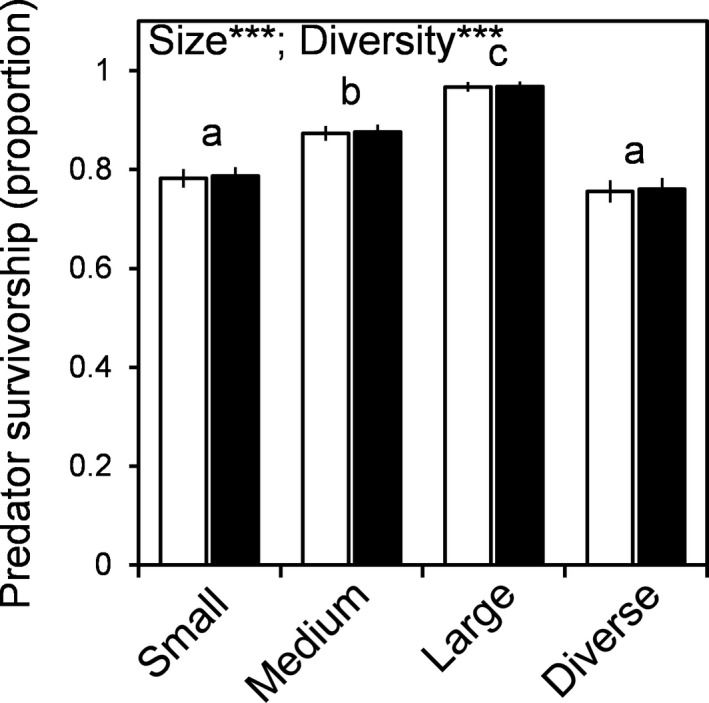
Influences of predator size‐structure and species identity (*Panopeus *= white; *Eurytium* = black) on mean predator survivorship recorded on seven occasions during the experiment. Means and standard errors are model estimates (on the response scale) based on the GLMM. Letters denote results of post hoc Tukey tests for size‐structure (across species) in the absence of a size*identity effect. Significant factors are shown (***p < 0.001). Error bars are 1*SE* of the mean

Predator survivorship improved with increasing body size (means: *S* = 78%, *M* = 87%, *L* = 96%; *χ* = 99.7, *p* < 0.001; Figure 1; Supporting Information Appendix S3), but was unaffected by species identity (*χ* = 0.033, *p* = 0.856). As expected under size‐based cannibalism, survivorship declined with body size diversity (single: 88%, diverse: 76%; *z* = 6.22, *p* < 0.001; Figure 1).

### 3.2 Overall effects of predators

**Figure 2:**
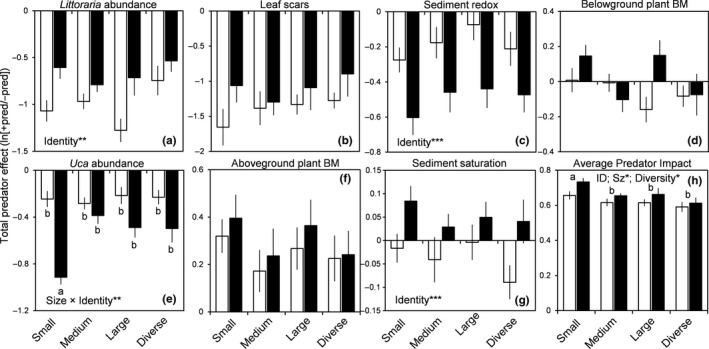
Influences of predator size‐structure and species identity (*Panopeus *= white; *Eurytium* = black) on the strength of total predator effects on individual prey, sediment, and plant response variables (a–g), as well as the average predator impact (API, a measure of multifunctionality; h) across these variables. Letters denote results of post hoc Tukey tests, for all treatments in the presence of a size*identity effect (e) or for size‐structure (across species) in the absence of a size*identity effect (h). Significant factor(s) are shown in each panel; the level of significance is included (after FDR correction; ***p < 0.001, **p < 0.01; *p < 0.05). In h, ID = identity; Sz = size; * indicates ΔAIC >3 for main factors, or *p* < 0.05 for treatment or diversity contrasts. Error bars are 1*SE* of the mean

The presence of predators broadly affected prey, sediment, and plant response variables. Across size treatments and species, predators suppressed *Littoraria* abundance by 53% (Figure 2a; *p* < 0.001) and *Uca* abundance by 31% (Figure 2b; *p* < 0.001). Predators also reduced the impacts of *Littoraria* on leaves (leaf scars) by 20% (Figure 2c; *p* < 0.001), elevated aboveground *Spartina* biomass by 27% (Figure 2d; *p* < 0.001), and caused a decline in sediment redox potential (Figure 2e; *p* < 0.001), but did not affect sediment saturation (Figure 2f; *p* = 0.619) or belowground *Spartina* biomass (Figure 2g; *p* = 0.639; Supporting Information Appendix S4).

### 3.3 Size‐structure and total predator effects

Size class strongly interacted with species identity to determine *Uca* abundance, with small *Eurytium* leading to anomalously low abundance of these engineers (Figure 2b, size*species: *F*_3,56_ = 4.905, *p* < 0.016). Moreover, API was altered by size‐structure (Figure 2h; ΔAIC = −7.81), irrespective of species identity (species*size: ΔAIC = 4.30). This was due to small predators collectively having a greater API than medium (*z* = 2.61; *p* = 0.027), large (*z* = 2.36, *p* = 0.036), and diverse (*z* = 3.91; *p* < 0.001) predators. API was also generally reduced by predator diversity (Figure 2h, *z* = −2.81; *p* = 0.014). Finally, *Eurytium* led to greater *Littoraria* abundance (Figure 2a; *F*_1,56_ = 15.28, *p* = 0.002), lower sediment redox potential (Figure 2e; *F*_1,56_ = 53.412, *p* < 0.001), lower sediment saturation (Figure 2f; *F*_1,56_ = 11.250, *p* = 0.013), and greater API (Figure 2h; ΔAIC = −5.03).

## 4 DISCUSSION

Our results show that resident predatory crabs strongly and broadly affect ecosystem functioning in our southeastern U.S. salt marsh study system and that predator size‐structure and identity have the potential to modify these effects. We report that size diversity, and an associated increase in cannibalism, slightly weakened the multifunctional impacts of predators. Although most effects of size‐structure were weak, in one predator species, a shift to smaller predators dramatically strengthened trophic control of an important ecosystem engineer. Collectively, these results show that, although many of the functional contributions of predators may exhibit some resistance to size shifts, others may be highly—and surprisingly—sensitive.

As stated above, one of our most striking results was that the strong effect of predator size class on trophic control of a functionally important prey species, and the fact this was contingent on predator identity. Specifically, size strongly interacted with species identity to determine the abundance of *Uca*, a key ecosystem engineer known to improve sediment aeration and plant growth in salt marshes (Bertness, 1985). While this interactive effect did not further cascade in our experiment, propagation of such impacts may emerge over longer periods or in the face of high grazer densities (Gittman & Keller, 2013). Overall, this unexpected result adds to a growing recognition that shifts in predator body size can have variable impacts across species (Rudolf & Rasmussen, 2013b; Rudolf et al., 2014), even where they appear to be functionally redundant (see Section 1). Therefore, shifting the mean size of predators, through processes such as harvesting and climate change, may unleash unforeseen, species‐specific outcomes at the ecosystem level.

Our results also provide the first evidence that size diversity can non‐additively buffer predators’ multifunctional impacts. This effect likely emanated from the increased predator mortality rates in diverse size treatments, consistent with larger predators using their size advantage to trophically exploit smaller conspecifics (Griffen & Byers, 2006; Rudolf, 2007). This would have caused—albeit temporary, between replenishments—density reductions and likely induced density‐independent fear effects on the activity of smaller predators. Had we allowed longer‐term declines in predator density during the experiment, the negative effects of predator size diversity on their multifunctional impacts would most likely have been greater. Although intraspecific size diversity probably also increases complementary use of shared resources (Ye, Chang, Garcia‐Comas, Gong, & Hsieh, 2013), our study highlights the modest yet consistent multifunctional effects when cannibalism prevails. Losses of size diversity, independent of mean size, may therefore act to strengthen ecosystem impacts of predators, which could be destabilizing to food webs (McCann, Hastings, & Huxel, 1998) and become a management concern with respect to invasive (e.g., bivalve‐eating green crabs; Miron, Audet, Landry, & Moriyasu, ) or native (e.g., coral‐eating gastropods; Burkepile & Hay, 2007) mesopredators.

Notwithstanding the above‐described effects, the majority of response variables were resistant to shifts in predator size‐structure. This can be explained by the greater numerical abundance (and approximate energetic equivalence) of smaller predators, an attribute of our experiment designed to reflect commonly observed size‐abundance scaling (Damuth, 1981). It reiterates that the relevance of traits for ecosystem functions is mediated by the abundance of organisms possessing them (Grime, 1998). Importantly, small predators did not appear to be limited by (claw) gape when faced with focal prey (pers. obs.). Despite per capita differences, therefore, the collective ecological functions of predators were largely resistant to considerable shifts in their mean size (fivefold variation in individual mass) and in some cases (e.g., API of small vs. medium, large, or diverse predators) were even strengthened by a shift to smaller individuals. But ecologists and environmental managers must be wary before assuming functional resistance in the face of size shifts, as the effects of size‐structure on predators’ multifunctional impacts and regulation of ecosystem engineers illustrate.

Species identity had broad independent functional consequences that spanned prey (i.e., *Littoraria*) and sediment responses (i.e., saturation, redox), as well as our integrative measure of predator impact (i.e., API). We suspect a strength–speed trade‐off is in evidence, with stronger‐clawed *Panopeus* better equipped to take advantage of slow‐moving, hard‐shelled, *Littoraria*, and faster‐moving *Eurytium* better able to exploit rapid, though weaker shelled, *Uca* (Griffin et al., 2015). Unexpectedly, the predator species also seem to differ in their burrowing behavior: Our casual field observations suggest that *Eurytium* more actively maintains its burrows, bringing water‐logged sediment to the surface and potentially helping to explain the effects of *Eurytium* of sediment saturation and redox. These results reinforce that, even when predator species are indistinguishable across commonly assessed trait categories (e.g., size, foraging mode, and habitat domain), they may still differ substantially in their ecosystem impacts (Resetarits & Chalcraft, 2007), possibly attributable to more fine‐scale traits, for example, biomechanical traits related to foraging.

Finally, our results have implications for salt marsh ecology by revealing the broad functional impacts of resident predators. Importantly, our experimental results demonstrate that the focal predators, common in southeastern U.S. marshes, suppress the strongly interacting grazer, *Littoraria*, and, for the first time, show that they facilitate the ecosystem's foundation species, *Spartina*. These predators also suppress ecosystem engineering fiddler crabs and lower sediment redox potential, long known to be a key indicator of microbial function, decomposition, and nutrient cycling in coastal wetland soils (e.g., Hackney, 1987, Gribsholt et al., 2003). Our results therefore call for the incorporation of resident predators into biogeochemical models of salt marshes; they also highlight where consideration of predator size‐structure and identity would refine such models.

In summary, experimental shifts in predator size‐structure modified the functional roles of salt marsh predators in subtle, and occasionally unpredictable, ways. Thus, although size is recognized as a key functional trait, its influence may not translate strongly to the ecosystem level, where abundance is an additional driver. However, the “devil is in the detail”: Despite the close taxonomic and functional relatedness of the predator species, within one of them, size shifts strongly affected the trophic control of ecosystem engineering prey. Overall, our study shows that, although the ecosystem contributions of predators may show some resistance to species turnover and shifts in size‐structure, they may also respond in surprising ways that are challenging to predict through trait‐based ecology.

## AUTHOR CONTRIBUTIONS

JG and BS conceived and designed the study; JG analyzed the data with inputs from BS; JG wrote the paper with inputs from BS; JG and BS agree to be accountable for all aspects of the work.

## DATA ACCESSIBILITY

Data are available through Figshare: https://figshare.com/articles/Predator_size-structure_and_species_identity_determine_cascading_effects_in_a_coastal_ecosystem/6989432.

## References

[ece34571-bib-0001] Angelini, C. , van der Heide, T. , Griffin, J. N. , Morton, J. P. , Derksen‐Hooijberg, M. , Lamers, L. P. M. , … Silliman, B. R. (2015). Foundation species' overlap enhances biodiversity and multifunctionality from the patch to landscape scale in southeastern United States salt marshes. Proceedings of the Royal Society B: Biological Sciences, 282(1811), 20150421 10.1098/rspb.2015.0421 PMC4528541 26136442

[ece34571-bib-0002] Bates, D. , Maechler, M. , Bolker, B. M. , & Walker, S. C. (2015). Fitting linear mixed‐effects models using lme4. Journal of Statistical Software, 67, 1–48.

[ece34571-bib-0003] Benjamini, Y. , & Hochberg, Y. (1995). Controlling the false discovery rate – A practical and powerful approach to multiple testing. Journal of the Royal Statistical Society Series B‐Methodological, 57, 289–300.

[ece34571-bib-0004] Berlow, E. L. (1999). Strong effects of weak interactions in ecological communities. Nature, 398, 330 10.1038/18672

[ece34571-bib-0005] Bertness, M. D. (1985). Fiddler crab regulation of spartina‐alterniflora production on a New‐England salt‐marsh. Ecology, 66, 1042–1055. 10.2307/1940564

[ece34571-bib-0006] Brose, U. (2010). Body‐mass constraints on foraging behaviour determine population and food‐web dynamics. Functional Ecology, 24, 28–34. 10.1111/j.1365-2435.2009.01618.x

[ece34571-bib-0007] Burkepile, D. E. , & Hay, M. E. (2007). Predator release of the gastropod *Cyphoma gibbosum* increases predation on gorgonian corals. Oecologia, 154, 167–173. 10.1007/s00442-007-0801-4 17636334

[ece34571-bib-0008] Byrnes, J. E. K. , Gamfeldt, L. , Isbell, F. , Lefcheck, J. S. , Griffin, J. N. , Hector, A. , … Duffy, J. E. (2014). Investigating the relationship between biodiversity and ecosystem multifunctionality: Challenges and solutions. Methods in Ecology and Evolution, 5, 111–124. 10.1111/2041-210X.12143

[ece34571-bib-0009] Chalcraft, D. R. , & Resetarits, W. J. (2004). Metabolic rate models and the substitutability of predator populations. Journal of Animal Ecology, 73, 323–332. 10.1111/j.0021-8790.2004.00809.x

[ece34571-bib-0010] Cribari‐Neto, F. , & Zeileis, A. (2010). Beta regression in R. Journal of Statistical Software, 34, 1–24.

[ece34571-bib-0011] Damuth, J. (1981). Population‐density and body size in mammals. Nature, 290, 699–700. 10.1038/290699a0

[ece34571-bib-0012] Dirzo, R. , Young, H. S. , Galetti, M. , Ceballos, G. , Isaac, N. J. B. , & Collen, B. (2014). Defaunation in the Anthropocene. Science, 345, 401–406. 10.1126/science.1251817 25061202

[ece34571-bib-0013] Duffy, J. E. (2003). Biodiversity loss, trophic skew and ecosystem functioning. Ecology Letters, 6, 680–687. 10.1046/j.1461-0248.2003.00494.x

[ece34571-bib-0014] Duffy, J. E. , Richardson, J. P. , & Canuel, E. A. (2003). Grazer diversity effects on ecosystem functioning in seagrass beds. Ecology Letters, 6, 881–881.

[ece34571-bib-0015] Emmerson, M. C. , & Raffaelli, D. (2004). Predator‐prey body size, interaction strength and the stability of a real food web. Journal of Animal Ecology, 73, 399–409. 10.1111/j.0021-8790.2004.00818.x

[ece34571-bib-0016] Gardner, J. L. , Peters, A. , Kearney, M. R. , Joseph, L. , & Heinsohn, R. (2011). Declining body size: A third universal response to warming? Trends in Ecology & Evolution, 26, 285–291. 10.1016/j.tree.2011.03.005 21470708

[ece34571-bib-0017] Gittman, R. K. , & Keller, D. A. (2013). Fiddler crabs facilitate *Spartina alterniflora* growth, mitigating periwinkle overgrazing of marsh habitat. Ecology, 94, 2709–2718. 24597218 10.1890/13-0152.1

[ece34571-bib-0018] Gribsholt, B. , Kostka, J. E. , & Kristensen, E. (2003). Impact of fiddler crabs and plant roots on sediment biogeochemistry in a Georgia saltmarsh. Marine Ecology Progress Series, 259, 237–251. 10.3354/meps259237

[ece34571-bib-0019] Griffen, B. D. , & Byers, J. E. (2006). Intraguild predation reduces redundancy of predator species in multiple predator assemblage. Journal of Animal Ecology, 75, 959–966. 10.1111/j.1365-2656.2006.01115.x 17009759

[ece34571-bib-0020] Griffen, B. D. , & Mosblack, H. (2011). Predicting diet and consumption rate differences between and within species using gut ecomorphology. Journal of Animal Ecology, 80, 854–863. 10.1111/j.1365-2656.2011.01832.x 21418211

[ece34571-bib-0021] Griffin, J. N. , Byrnes, J. E. K. , & Cardinale, B. J. (2013). Effects of predator richness on prey suppression: A meta‐analysis. Ecology, 94, 2180–2187. 10.1890/13-0179.1 24358704

[ece34571-bib-0022] Griffin, J. N. , Toscano, B. J. , Griffen, B. D. , & Silliman, B. R. (2015). Does relative abundance modify multiple predator effects? Basic and Applied Ecology, 16, 641–651. 10.1016/j.baae.2015.05.003

[ece34571-bib-0023] Grime, J. P. (1998). Benefits of plant diversity to ecosystems: Immediate, filter and founder effects. Journal of Ecology, 86, 902–910. 10.1046/j.1365-2745.1998.00306.x

[ece34571-bib-0024] Hackney, C. T. (1987). Factors affecting accumulation or loss of macroorganic matter in salt‐marsh sediments. Ecology, 68, 1109–1113. 10.2307/1938385

[ece34571-bib-0025] Holdredge, C. , Bertness, M. D. , Herrmann, N. C. , & Gedan, K. B. (2010). Fiddler crab control of cordgrass primary production in sandy sediments. Marine Ecology Progress Series, 399, 253–259. 10.3354/meps08331

[ece34571-bib-0026] Hothorn, T. , Bretz, F. , & Westfall, P. (2008). Simultaneous inference in general parametric models. Biometrical Journal, 50, 346–363. 18481363 10.1002/bimj.200810425

[ece34571-bib-0027] Jochum, M. , Schneider, F. D. , Crowe, T. P. , Brose, U. , & O'Gorman, E. J. (2012). Climate‐induced changes in bottom‐up and top‐down processes independently alter a marine ecosystem. Philosophical Transactions of the Royal Society B‐Biological Sciences, 367, 2962–2970. 10.1098/rstb.2012.0237 PMC3479746 23007084

[ece34571-bib-0028] Kleiber, M. (1932). Body size and metabolism. Hilgardia, 6, 315–332. 10.3733/hilg.v06n11p315

[ece34571-bib-0029] Krenek, L. , & Rudolf, V. H. W. (2014). Allometric scaling of indirect effects: Body size ratios predict non‐consumptive effects in multi‐predator systems. Journal of Animal Ecology, 83, 1461–1468. 10.1111/1365-2656.12254 24910170

[ece34571-bib-0030] Lester, S. E. , Halpern, B. S. , Grorud‐Colvert, K. , Lubchenco, J. , Ruttenberg, B. I. , Gaines, S. D. , … Warner, R. R. (2009). Biological effects within no‐take marine reserves: A global synthesis. Marine Ecology Progress Series, 384, 33–46. 10.3354/meps08029

[ece34571-bib-0031] McCann, K. , Hastings, A. , & Huxel, G. R. (1998). Weak trophic interactions and the balance of nature. Nature, 395, 794–798. 10.1038/27427

[ece34571-bib-0032] McCauley, D. J. , Pinsky, M. L. , Palumbi, S. R. , Estes, J. A. , Joyce, F. H. , & Warner, R. R. (2015). Marine defaunation: Animal loss in the global ocean. Science, 347 10.1126/science.1255641 25593191

[ece34571-bib-0033] McElroy, D. J. , O'Gorman, E. J. , Schneider, F. D. , Hetjens, H. , Le Merrer, P. , Coleman, R. A. , & Emmerson, M. (2015). Size‐balanced community reorganization in response to nutrients and warming. Global Change Biology, 21, 3971–3981. 10.1111/gcb.13019 26147063

[ece34571-bib-0034] Miron, G. , Audet, D. , Landry, T. , & Moriyasu, M. (2005). Predation potential of the invasive green crab (*Carcinus maenas*) and other common predators on commercial bivalve species found on Prince Edward island. Journal of Shellfish Research, 24, 579–586.

[ece34571-bib-0035] Northfield, T. D. , Snyder, G. B. , Ives, A. R. , & Snyder, W. E. (2010). Niche saturation reveals resource partitioning among consumers. Ecology Letters, 13, 338–348. 10.1111/j.1461-0248.2009.01428.x 20455919

[ece34571-bib-0036] Petchey, O. L. , & Belgrano, A. (2010). Body‐size distributions and size‐spectra: Universal indicators of ecological status? Biology Letters, 6, 434–437. 10.1098/rsbl.2010.0240 20444761 PMC2936225

[ece34571-bib-0037] Resetarits, W. J. , & Chalcraft, D. R. (2007). Functional diversity within a morphologically conservative genus of predators: Implications for functional equivalence and redundancy in ecological communities. Functional Ecology, 21, 793–804. 10.1111/j.1365-2435.2007.01282.x

[ece34571-bib-0038] Rudolf, V. H. W. (2007). Consequences of stage‐structured predators: Cannibalism, behavioral effects, and trophic cascades. Ecology, 88, 2991–3003. 10.1890/07-0179.1 18229834

[ece34571-bib-0039] Rudolf, V. H. W. (2012). Seasonal shifts in predator body size diversity and trophic interactions in size‐structured predator‐prey systems. Journal of Animal Ecology, 81, 524–532. 10.1111/j.1365-2656.2011.01935.x 22191419

[ece34571-bib-0040] Rudolf, V. H. W. , & Rasmussen, N. L. (2013a). Ontogenetic functional diversity: Size structure of a keystone predator drives functioning of a complex ecosystem. Ecology, 94, 1046–1056. 23858645 10.1890/12-0378.1

[ece34571-bib-0041] Rudolf, V. H. W. , & Rasmussen, N. L. (2013b). Population structure determines functional differences among species and ecosystem processes. Nature Communications, 4, 2318. 10.1038/ncomms3318 23933614

[ece34571-bib-0042] Rudolf, V. H. W. , Rasmussen, N. L. , Dibble, C. J. , & Van Allen, B. G. (2014). Resolving the roles of body size and species identity in driving functional diversity. Proceedings of the Royal Society B: Biological Sciences, 281(1781), 20133203 10.1098/rspb.2013.3203 PMC3953845 24598423

[ece34571-bib-0043] Schmitz, O. J. (2008). Effects of predator hunting mode on grassland ecosystem function. Science, 319, 952–954. 10.1126/science.1152355 18276890

[ece34571-bib-0044] Schmitz, O. J. , & Price, J. R. (2011). Convergence of trophic interaction strengths in grassland food webs through metabolic scaling of herbivore biomass. Journal of Animal Ecology, 80, 1330–1336. 10.1111/j.1365-2656.2011.01882.x 21722105

[ece34571-bib-0045] Shackell, N. L. , Frank, K. T. , Fisher, J. A. D. , Petrie, B. , & Leggett, W. C. (2010). Decline in top predator body size and changing climate alter trophic structure in an oceanic ecosystem. Proceedings of the Royal Society B‐Biological Sciences, 277, 1353–1360. 10.1098/rspb.2009.1020 PMC2871930 20031989

[ece34571-bib-0046] Silliman, B. R. , Layman, C. A. , Geyer, K. , & Zieman, J. C. (2004). Predation by the black‐clawed mud crab, Panopeus herbstii, in Mid‐Atlantic salt marshes: Further evidence for top‐down control of marsh grass production. Estuaries, 27, 188–196. 10.1007/BF02803375

[ece34571-bib-0047] Silliman, B. R. , & Zieman, J. C. (2001). Top‐down control of *Spartina alterniflora* production by periwinkle grazing in a Virginia salt marsh. Ecology, 82, 2830–2845. 10.2307/2679964

[ece34571-bib-0048] Solan, M. , Cardinale, B. J. , Downing, A. L. , Engelhardt, K. A. M. , Ruesink, J. L. , & Srivastava, D. S. (2004). Extinction and ecosystem function in the marine benthos. Science, 306, 1177–1180. 10.1126/science.1103960 15539601

[ece34571-bib-0049] Toscano, B. J. , & Griffen, B. D. (2012). Predatory crab size diversity and bivalve consumption in oyster reefs. Marine Ecology Progress Series, 445, 65–74. 10.3354/meps09461

[ece34571-bib-0050] Woodward, G. , Ebenman, B. , Emmerson, M. , Montoya, J. M. , Olesen, J. M. , Valido, A. , & Warren, P. H. (2005). Body size in ecological networks. Trends in Ecology & Evolution, 20, 402–409. 10.1016/j.tree.2005.04.005 16701403

[ece34571-bib-0051] Ye, L. , Chang, C.‐Y. , Garcia‐Comas, C. , Gong, G.‐C. , & Hsieh, C.‐H. (2013). Increasing zooplankton size diversity enhances the strength of top‐down control on phytoplankton through diet niche partitioning. Journal of Animal Ecology, 82, 1052–1060. 10.1111/1365-2656.12067 23506226

