# Supplementary material for: Predator size‐structure and species identity determine cascading effects in a coastal ecosystem
Source: Ecol Evol. 2018 Dec 1;8(24):12435–42. doi: 10.1002/ece3.4571 (PMC6308854; doi:10.1002/ece3.4571)
Supplement: Supplementary file 1 [file ECE3-8-12435-s001.docx]

**Supplementary materials**

**Appendix S1. Schematic of experimental design.**

**Figure S1**. Schematic of the experimental design. Boxes show the treatments, size-structure varies horizontally, and species identity vertically. Photos are of representative individuals within each size class and are to scale relative to one another. See main text for size classes. Numerical abundances set in the experiment were as shown in each treatment box.

**Appendix S2. Proportion of females (sex ratio) across experimental treatments.**

**Figure S1**. Variation in the proportion of females across treatments of predator size-structure and species identity (*Panopeus* = white; *Eurytium* = black) (based on data gathered during establishment of the experiment).

**Table S1.** Analysis of the proportion of females across treatment, based on a two-way analysis of variance.

| Source |  |  |  |  |  |
| --- | --- | --- | --- | --- | --- |
|  | DF | SS | MS | F | P |
| Size | 3 | 0.10714 | 0.52435 | 9.6233 | **<0.001** |
| Species | 1 | 0.55213 | 0.55213 | 10.1331 | **0.002378** |
| Size*Species | 3 | 0.83001 | 0.27667 | 5.0776 | **0.003519** |
| Residuals | 56 | 3.015131 | 0.05449 |  |  |

**Appendix S3. Effects of predator size-structure and identity on predator survivorship: statistical summary.**

**Table S1**. Summary of fixed effects in the generalized linear mixed model testing the effects of predator size-structure (small, medium, large, diverse) and identity (*Panopeus*, *Eurytium*) on predator survivorship during the field experiment. Enclosure ID was included as a random effect (effect not shown) to account for repeated samples through time (approximately biweekly during the experiment). The intercept represents the treatment of Identity = *Panopeus*, Size = small.

| Fixed Effects |  |  |  |  |
| --- | --- | --- | --- | --- |
|  | Coeff. estimate | Std. Error | z-value | Pr(>\|z\|) |
| (intercept) | 1.31858 | 0.12612 | 10.455 | <0.001 |
| Medium | 0.58458 | 0.21464 | 2.724 | 0.00646 |
| Large | 2.70385 | 0.59957 | 4.510 | <0.001 |
| Diverse | -0.28551 | 0.19255 | -1.483 | 0.13813 |
| Euytium | -0.05779 | 0.17708 | -0.326 | 0.74417 |
| Medium:Eurytium | 0.13922 | 0.30556 | 0.456 | 0.64865 |
| Large:Eurytium | -0.95490 | 0.71434 | -1.337 | 0.18130 |
| Diverse:Eurytium | 0.28232 | 0.27534 | 1.025 | 0.30519 |

**Table S2**: Overall effects of predator size and identity based on the model reported in Table S1 above. Effects were established by dropping parameters associated with each factor separately from the complete model. AIC (none) = Akaike Information Criterion of the complete model; AIC (drop1) = AIC of the model when the respective factor is dropped; LRT = likelihood ratio test.

| Factors |  |  |  |  |  |
| --- | --- | --- | --- | --- | --- |
|  | DF | AIC (none) | AIC (drop1) | LRT | *P* |
| Size | 3 | 1123 | 1190 | 72.981 | **<0.001** |
| Species | 1 | 1123 | 1121 | 0.053 | 0.817 |
| Species*Size | 3 | 1125.3 | 1123.0 | 3.6456 | 0.302 |

**Appendix S4. Results of ANOVAs on Predator Effects.**

The tables below show the results of two-way ANOVAs investigating the effects of size-structure and species identity on total predator effects. One table is presented for each response variable.

Note that in the following Tables, the factor ‘size-structure’ (4 levels: small, medium, large, diverse) is abbreviated to ‘size’, and the factor ‘species identity’ (2 levels: *Panopeus*, *Eurytium*) is abbreviated to ‘species’. In these models, since the intercept represents the grand mean, it can be interpreted as the general effect of predators on a response variable. P-values used to evaluate stated predictions (size, species, size*species) are presented as FDR-corrected values. P<0.001 is presented as .000.

| **Table S1. Total predator effect (PE_t_) on *Littoraria*.** | | | | | |
| --- | --- | --- | --- | --- | --- |
|  | | | | | |
| Source | Type III Sum of Squares | df | Mean Square | F | Sig. |
| Corrected Model | 3.478^a^ | 7 | .497 | 3.802 | .002 |
| Intercept | 44.937 | 1 | 44.937 | 343.933 | .000 |
| size | 1.051 | 3 | .350 | 2.683 | .132 |
| species | 1.996 | 1 | 1.996 | 15.280 | .002 |
| size * species | .430 | 3 | .143 | 1.096 | .508 |
| Error | 7.317 | 56 | .131 |  |  |
| Total | 55.732 | 64 |  |  |  |
| Corrected Total | 10.794 | 63 |  |  |  |
| 1. R Squared = .322 (Adjusted R Squared = .237)  \| **Table S2. Total predator effect (PE_t_) on *Uca*.** \| \| \| \| \| \| \| --- \| --- \| --- \| --- \| --- \| --- \| \|  \| \| \| \| \| \| \| Source \| Type III Sum of Squares \| df \| Mean Square \| F \| Sig. \| \| Corrected Model \| 3.050^a^ \| 7 \| .436 \| 9.305 \| .000 \| \| Intercept \| 10.635 \| 1 \| 10.635 \| 227.134 \| .000 \| \| size \| .639 \| 3 \| .213 \| 4.547 \| .023 \| \| species \| 1.722 \| 1 \| 1.722 \| 36.783 \| .000 \| \| size * species \| .689 \| 3 \| .230 \| 4.905 \| .016 \| \| Error \| 2.622 \| 56 \| .047 \|  \|  \| \| Total \| 16.306 \| 64 \|  \|  \|  \| \| Corrected Total \| 5.672 \| 63 \|  \|  \|  \| \| a. R Squared = .538 (Adjusted R Squared = .480) \| \| \| \| \| \| | | | | | |

**Table S3. Total predator effect (PE_t_) on leaf scars.**

|  | | | | | |
| --- | --- | --- | --- | --- | --- |
|  | | | | | |
| Source | Type III Sum of Squares | df | Mean Square | F | Sig. |
| Corrected Model | 3.010^a^ | 7 | .430 | .937 | .486 |
| Intercept | 99.933 | 1 | 99.933 | 217.620 | .000 |
| size | .774 | 3 | .258 | .562 | .768 |
| species | 1.686 | 1 | 1.686 | 3.672 | .137 |
| size * species | .550 | 3 | .183 | .399 | .836 |
| Error | 25.716 | 56 | .459 |  |  |
| Total | 128.659 | 64 |  |  |  |
| Corrected Total | 28.726 | 63 |  |  |  |
| a. R Squared = .105 (Adjusted R Squared = -.007) | | | | | |

**Table S4. Total predator effect (PE_t_) on Sediment saturation.**

|  | | | | | |
| --- | --- | --- | --- | --- | --- |
|  | | | | | |
| Source | Type III Sum of Squares | df | Mean Square | F | Sig. |
| Corrected Model | .173^a^ | 7 | .025 | 2.220 | .046 |
| Intercept | .003 | 1 | .003 | .250 | .619 |
| size | .034 | 3 | .011 | 1.019 | .531 |
| species | .125 | 1 | .125 | 11.250 | .007 |
| size * species | .014 | 3 | .005 | .411 | .836 |
| Error | .623 | 56 | .011 |  |  |
| Total | .799 | 64 |  |  |  |
| Corrected Total | .796 | 63 |  |  |  |
| a. R Squared = .217 (Adjusted R Squared = .119) | | | | | |

| **Table S5. Total predator effect (PE_t_) on Sediment redox potential.** | | | | | |
| --- | --- | --- | --- | --- | --- |
|  | | | | | |
| Source | Type III Sum of Squares | df | Mean Square | F | Sig. |
| Corrected Model | 1.837^a^ | 7 | .262 | 9.140 | .000 |
| Intercept | 7.336 | 1 | 7.336 | 255.485 | .000 |
| size | .277 | 3 | .092 | 3.219 | .082 |
| species | 1.534 | 1 | 1.534 | 53.412 | .000 |
| size * species | .026 | 3 | .009 | .303 | .897 |
| Error | 1.608 | 56 | .029 |  |  |
| Total | 10.781 | 64 |  |  |  |
| Corrected Total | 3.445 | 63 |  |  |  |
| a. R Squared = .533 (Adjusted R Squared = .475) | | | | | |

| **Table S6. Total predator effect (PE_t_) on aboveground plant biomass.** | | | | | |
| --- | --- | --- | --- | --- | --- |
|  | | | | | |
| Source | Type III Sum of Squares | df | Mean Square | F | Sig. |
| Corrected Model | .321^a^ | 7 | .046 | .609 | .746 |
| Intercept | 4.935 | 1 | 4.935 | 65.539 | .000 |
| size | .243 | 3 | .081 | 1.076 | .509 |
| species | .064 | 1 | .064 | .848 | .509 |
| size * species | .014 | 3 | .005 | .062 | .995 |
| Error | 4.216 | 56 | .075 |  |  |
| Total | 9.472 | 64 |  |  |  |
| Corrected Total | 4.537 | 63 |  |  |  |
| a. R Squared = .071 (Adjusted R Squared = -.045) | | | | | |

**Table S7. Total predator effect (PE_t_) on belowground plant biomass.**

| Source | Type III Sum of Squares | df | Mean Square | F | Sig. |
| --- | --- | --- | --- | --- | --- |
| Corrected Model | .721^a^ | 7 | .103 | 1.469 | .197 |
| Intercept | .016 | 1 | .016 | .222 | .639 |
| size | .227 | 3 | .076 | 1.079 | .509 |
| species | .129 | 1 | .129 | 1.844 | .328 |
| size * species | .365 | 3 | .122 | 1.734 | .328 |
| Error | 3.929 | 56 | .070 |  |  |
| Total | 4.666 | 64 |  |  |  |
| Corrected Total | 4.650 | 63 |  |  |  |
| a. R Squared = .155 (Adjusted R Squared = .050) | | | | | |
